# Percutaneous ultrasonic debridement of tendinopathy—a pilot Achilles rabbit model

**DOI:** 10.1186/s13018-015-0207-7

**Published:** 2015-05-20

**Authors:** Srinath Kamineni, Timothy Butterfield, Anthony Sinai

**Affiliations:** Elbow Shoulder Research Centre, Department of Orthopaedics and Sports Medicine, University of Kentucky, Lexington, KY 40536 USA; Department of Athletic Training, University of Kentucky, Lexington, KY 40536 USA; Department of Microbiology, University of Kentucky, Lexington, KY 40536 USA

**Keywords:** Tendinopathy, Animal model, Ultrasonic treatment, Collagen, Histology, Collagenase

## Abstract

**Background:**

Tendinopathy is a common clinical pathology, with mixed treatment results, especially when chronic. In this study, we examine the effects of an ultrasonic debridement modality in a rabbit tendinopathy model.

We asked four questions: 1) Was it possible to create and visualize with ultrasound a tendinopathy lesion in a rabbit Achilles tendon? 2) Was it possible to guide a 19-gauge ultrasonic probe into the tendinopathy lesion? 3) Following ultrasonic treatment, was tendinopathy debris histologically present? and 4) Was the collagen profile qualitatively and quantitatively normalized following treatment?

**Methods:**

Skeletally mature female New Zealand white rabbits (*n* = 12) were injected with, ultrasonography localization, 0.150 ml of collagenase into the Achilles tendon. The collagenase-induced Achilles tendinopathy (3 weeks) was treated with percutaneous ultrasonic debridement. The tendons were harvested, at 3 weeks after treatment, and were subjected to histological assessment (modified Movin score) and biochemical analysis (collagen isoform content).

**Results:**

Histopathological examination revealed that all tendons injected with collagenase showed areas of hypercellularity and focal areas of tendon disorganization and degeneration. The treated tendons had lower (improved) histopathological scores than injured tendons (*P* < 0.001). Western blot analysis showed that ultrasonic therapy restored, within statistical limits, collagen type I, III, and X expressions in a treated tendon, to qualitative and semi-quantitative levels of a normal tendon.

**Conclusions:**

We were successfully able to create a collagenase-injected tendinopathy lesion in a rabbit Achilles tendon and visualize the lesion with an ultrasound probe. A 19-gauge ultrasonic probe was inserted into the tendinopathic lesion under direct ultrasound guidance, and minimal tendinopathic debris remained after treatment. The treated tendon demonstrated a normalized qualitative and semi-quantitative collagen profile and improved histological appearance in the short term. This technique demonstrates scientific merit with respect to the minimally invasive treatment of tendinopathy and warrants further studies.

**Clinical relevance:**

Recalcitrant tendinopathy has evaded consistent non-operative treatment since the tendinopathic debris remains in situ, to some extent, with non-operative approaches. This percutaneous emulsification/evacuation approach, under direct ultrasound visualization, has the potential to cure recalcitrant tendinopathies without open surgery, which would benefit the patient and result in significant healthcare cost reductions.

## Introduction

Tendinopathy is a widespread cause of morbidity affecting virtually all joints. Although the etiology is imperfectly understood, the path physiology is one of the degeneration and necrosis at the pathological site [1]. There are several treatment modalities currently used in clinical practice, including physical therapy, non-steroidal anti-inflammatories, and injections of corticosteroid and platelet-rich plasma (PRP) [2–4]. However, no universally accepted treatment is known to be completely safe and effective with a high degree of predictability.

In clinical situations, magnetic resonance imaging (MRI) and ultrasound scanning are commonly utilized to provide fine internal architectural details of symptomatic tendons, for diagnosis and evaluation of treatment [5]. Typically, ultrasound imaging of tendons has recently become a first-line investigation as it is widely available, relatively inexpensive, and is easy to use. In order to understand tendon biology and mechanics in normal and injury situations, a mouse model is commonly used [6, 7]. However, the use of diagnostic and localizing ultrasonography is less straightforward with mice than with a larger animal model, and hence, we chose a validated Achilles tendinopathy rabbit model [8, 9]. Furthermore, our choice of treatment is based on the previously published clinical use of ultrasonic energy to treat lateral epicondylitis [10].

The primary aim of this study was to characterize the mechanism by which ultrasonic emulsification and aspiration mechanistically produces its effect in a collagen degradation model. Our pilot study aims were to create a collagen degradation/tendinopathy rabbit model from literature data, treat the lesion using an ultrasonic aspiration probe, and analyze the results with histology and semi-quantification of the collagen profile. This study is the first step in characterizing the usefulness of ultrasonic treatment in tendinopathies, in a limited pilot form.

## Materials and methods

### Collagenase-induced injury

**Fig. 1:**
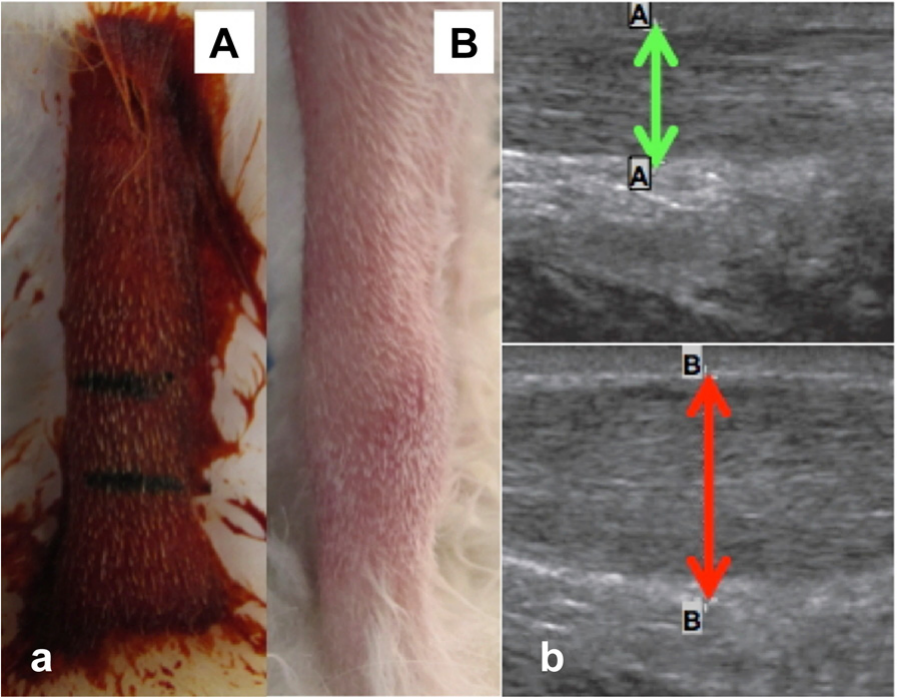
**a** (*A*) A normal betadine prepared rabbit Achilles tendon site. (*B*) An Achilles tendon at 3 weeks after collagenase injection demonstrating a fusiform swelling at the injection site. **b** (*A*) A normal Achilles tendon with a diameter *A*-*A*, (*B*) A collagenase-injected Achilles tendon after 3 weeks (group 1) with a significantly enlarged diameter *B*-*B*

The use of rabbits for experiments in this study was approved by the animal research ethics committee of the University of Kentucky. Twelve female New Zealand white rabbits (8 weeks old; weight, 2–2.5 kg) were used to create the tendinopathic model. Although we are aware of several models for the creation of a tendinopathy lesion, including chronic overload, prolonged PGE1 administration, etc., we chose the collagenase injection model as a method of studying collagen breakdown debris associated with tendinopathy [11–15]. They were randomly divided into control (group I) and treatment (group II) groups with isoflurane anesthesia utilized, and the scheme of treatment is shown in Table 1. One hundred and fifty microliters (10 mg/ml in 0.9 % saline) of collagenase I (Sigma-Aldrich, St Louis, MO, USA) was injected into the central region of the medial gastrocnemius part of the Achilles tendon (Fig. 1), 1 cm above the calcaneal tuberosity of the right limb of each rabbit under sterile conditions (Fig. 1), while the contralateral limb was left un-injected [8, 16]. The needle tip was localized to the center of the tendon under ultrasound guidance (GE E logic). Free cage activity, without any restrictions, was allowed after the collagenase injections. No adverse or unexpected morbidity or mortality was experienced during the test period. We additionally studied two rabbits (group 0) at 3 weeks after the collagenase injection, to confirm the development of a tendinopathic lesion, both by diagnostic ultrasound, and with histology and western blotting after harvesting the tissues. These were in addition to the group I and II specimens.

**Table 1:** Scheme of injection regimen and treatment with Tenex probe in rabbits

Group	Rabbit	Injected for (weeks)	Treated (weeks) retrieved [weeks]
0	1	3	No [3]
	2	3	No [3]
I	3	6	No [6]
	4	6	No [6]
	5	3	No [6]
	6	6	No [6]
II	7	3	3 [6]
	8	3	3 [6]
	9	3	3 [6]
	10	3	3 [6]
	11	3	3 [6]
	12	3	3 [6]
	13	3	3 [6]
	14	3	3 [6]

### Ultrasonic treatment

**Fig. 2:**
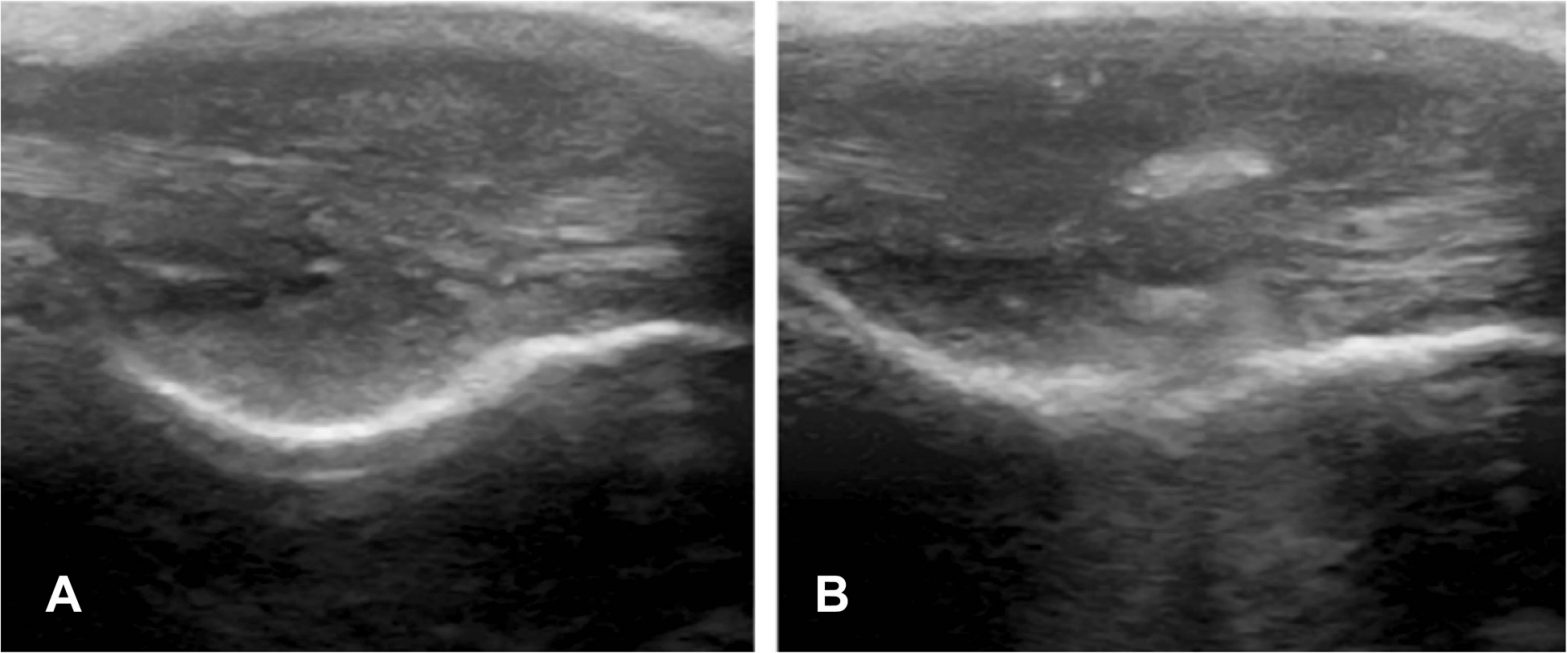
**a** Ultrasound scan of a rabbit Achilles tendon demonstrating a fusiform tendinopathic swelling and the internal tendon architecture 3 weeks after collagenase injection. **b** Ultrasound of the Achilles tendon demonstrating diffusion of the injection throughout the tendon

At week 3 after collagenase injection, group II rabbits were percutaneously treated with a Tenex™ ultrasonic probe (30 s), under ultrasound visualized guidance (Fig. 2), following which free cage activity was resumed. Group I and II rabbits were euthanased at 6 weeks, and the Achilles tendons were harvested for analysis; histological specimens were immediately preserved in formalin, and western blot samples were stored in liquid nitrogen and transferred to a −80 °C freezer. Group 0 was euthanased at 3 weeks.

### Histology

Histologic analysis [17] around the injection site was performed to determine the different responses of the tendon after the collagenase injection and the effectiveness of the treatment. The tendons were processed for histology, and 8-μm-thick sections were cut and stained with hematoxylin and eosin (H&E) [18–20]. The H&E slides were analyzed using a semi-quantitative histopathological grading scale: modified Movin score [21]. The score is based on eight parameters, with modifications based on the absence of minor parameters [8]. The parameters assessed were fiber structure and arrangement, rounding of nuclei, collagen stain ability, and four quadrant regional variations in cellularity. The assessment was based on multiple (*n* = 4) quadrant areas and averaged. The total score for a tendon could vary between 0 and 3 (Table 2).

**Table 2:** Scoring of histological sections to assess cellularity, vascularity, and collagen fiber organization

	Appearance	Description
Cellularity score		
0	Normal	Presence of flattened cells in a linear pattern between fibers
1	Slightly abnormal	Some rounded cells present, slight increase in cellularity
2	Abnormal	Many rounded cells present, obvious increase in cellularity
3	Markedly abnormal	Mostly rounded cells present, much higher numbers
Vascularity score		
0	Normal	Presence of some vascular bundles parallel to collagen fibers
1	Slightly abnormal	Slight increase in number of vascular bundles
2	Abnormal	Increased number of vascular bundles
3	Markedly abnormal	Large increase in number of vascular bundles
Organization score		
0	Normal	Parallel collagen fibers of similar widths
1	Slightly abnormal	Some loss of fiber organization, some loss of linearity
2	Abnormal	Moderate loss of fiber organization, few linear regions
3	Markedly abnormal	Total loss of organization, no linear fibers

Scoring was based on the system described by Movin et al. [23]

### Protein extraction

Tendons used for protein analysis were immediately stored in dry ice and subsequently in a −80° freezer. The tissue was homogenized using a 15-fold excess of extraction buffer (20 mM Tris, pH 7.4, 150 mM NaCl, 1 mM EDTA, 2 % SDS) and protease inhibitor cocktail (COMPLETE, Roche). The homogenate was centrifuged at 13,200 rpm for 30 min, and the supernatant was collected at −80 °C (Thermo Scientific Forma, −86 °C ULT Freezer, USA). Total protein concentration in the samples was determined using the BCA protein assay kit (Pierce, Thermo Scientific, USA).

### Western blot analysis

Protein levels for collagen types I, III, and X were assessed using western blot techniques. Aliquots of the protein samples were then boiled for 5 min in SDS sample buffer with 2-mercaptoethanol (Bio-Rad) as a reducing agent, and 10 μg of protein per lane was electrophoretically resolved on a 7 % SDS-PAGE and transferred to a nitrocellulose membrane (Pall, East Hill) using a semi-dry transfer cell apparatus (Trans-Blot SD, Bio-Rad, USA). Immunoblotting was done with anti-collagens I, III, and X antibodies (Abcam and Novus Biologicals, USA), used at 8 ng/ml for collagen I, 4 μg/ml for collagen III, and 500 ng/ml for collagen X. Calnexin in each sample was probed with rabbit anti-calnexin polyclonal antibody (2 μg/ml; Abcam, USA) as a loading control. Goat anti-rabbit IgG-perixodase (GE Healthcare, Piscataway, NJ, USA) was used as the secondary antibody at 1:8000 dilutions with ECL as the detection system (Fischer Scientific, USA). For each independent sample, immunoblotting was done in triplicate. For semi-quantification of western blot signals, the densities of specific antibodies and calnexin were measured with Image J (NIH, USA). The same-sized square was drawn around each band to measure the density, and background level near the band was subtracted from it. The levels of collagen subtype (I, III, and X) were normalized against calnexin levels.

### Statistical analysis

The ratios of treated to non-treated tendons were calculated for comparison. Student *t* test was used for statistical analysis. The contralateral Achilles tendons of the same rabbit were compared using Microsoft Excel (Microsoft Corporation, Seattle, WA, USA). Significance was set at a *P* value less than 0.05. We treated the Movin score as a continuous variable and used the Mann-Whitney *U* test. The assessments were descriptive and not analyzed statistically.

## Results

### Hematoxylin and eosin staining

**Fig. 3:**
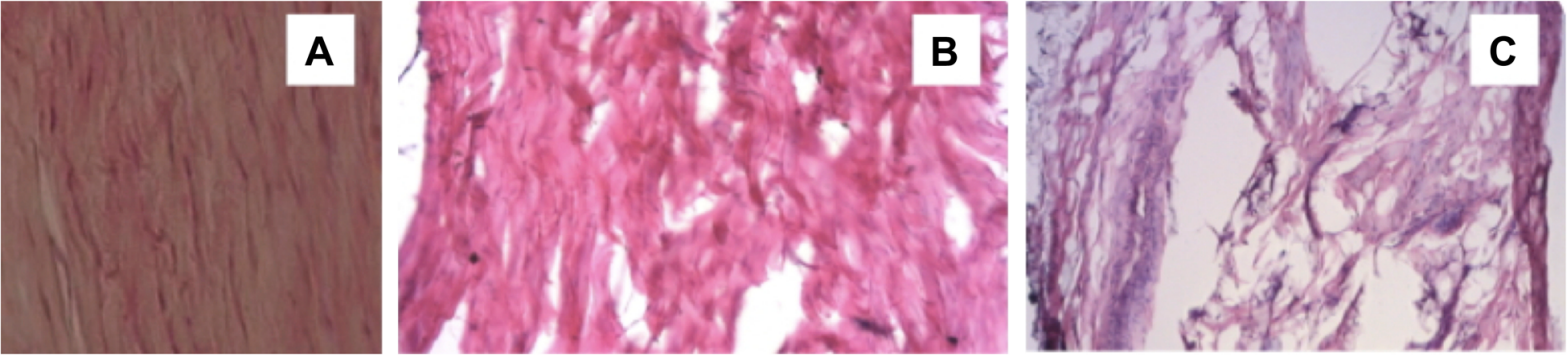
Staining of Achilles tendon sections with hematoxylin-eosin. **a** Normal Achilles tendon, **b** necrosis and disorganization induced by collagenase at 6 weeks, and **c** the histological appearance after 3 weeks of collagenase and 3 weeks of treatment reveals a repopulation of the evacuated cavity and early collagen bundles

Two pre-study pilot specimens (group 0) were analyzed at 3 weeks post-injection with collagenase, and the yielded mean pathologic sum score was 2.5 ± 0.577. The mean pathologic sum scores of the injured (group I—collagenase-induced pathological) tendons were greater than the mean pathologic score of the treated tendons (3.75 ± 0.5 versus 1.25 ± 0.462, *P* < 0.001) and control tendons (0.04 ± 0.294, *P* < 0.0007) (Table 3) (Fig. 3).

**Table 3:** Summary of pathologic scores of the control, injured, and treated tendons

Pathologic score	Contralateral controls	Injured tendons	Treated tendons
Mean	0.04	3.75	1.25
Median	0	3	1.5
SD	0.213	0.5	0.46

#### Assessment of each variable

Fiber arrangement: In the control tendons, the fibers were linear and parallel to each other, while collagenase-treated tendons showed complete disruption of linear fiber architecture. The fiber orientation of the ultrasonic-treated tendons was heterogeneous, since the normal peripheral fibers appeared ordered and linear, while the newly laid down fibers from the evacuation cavity were less ordered. The median for the control tendons was 0, injured tendons was 3 (group I), and for the ultrasonic-treated tendons was 1.5 (group II).

#### Cellularity

The randomly assigned four quadrants chosen for fiber orientation were analyzed for cellularity. Cellularity, based on nuclear staining, was observed to be dramatically increased (3/3) in the collagenase-treated group and increased (2/3) in the ultrasonic-treated group and normal in the controls (0/3).

#### Vascularity

Vascular bundles usually run parallel alongside the collagen fibers. The number of these vascular bundles increased with degeneration of the tendon. The median for the control tendon was 0, for the injured tendons was 3, and for the treated tendons was 1.5.

### Western blot analyses

**Fig. 4:**
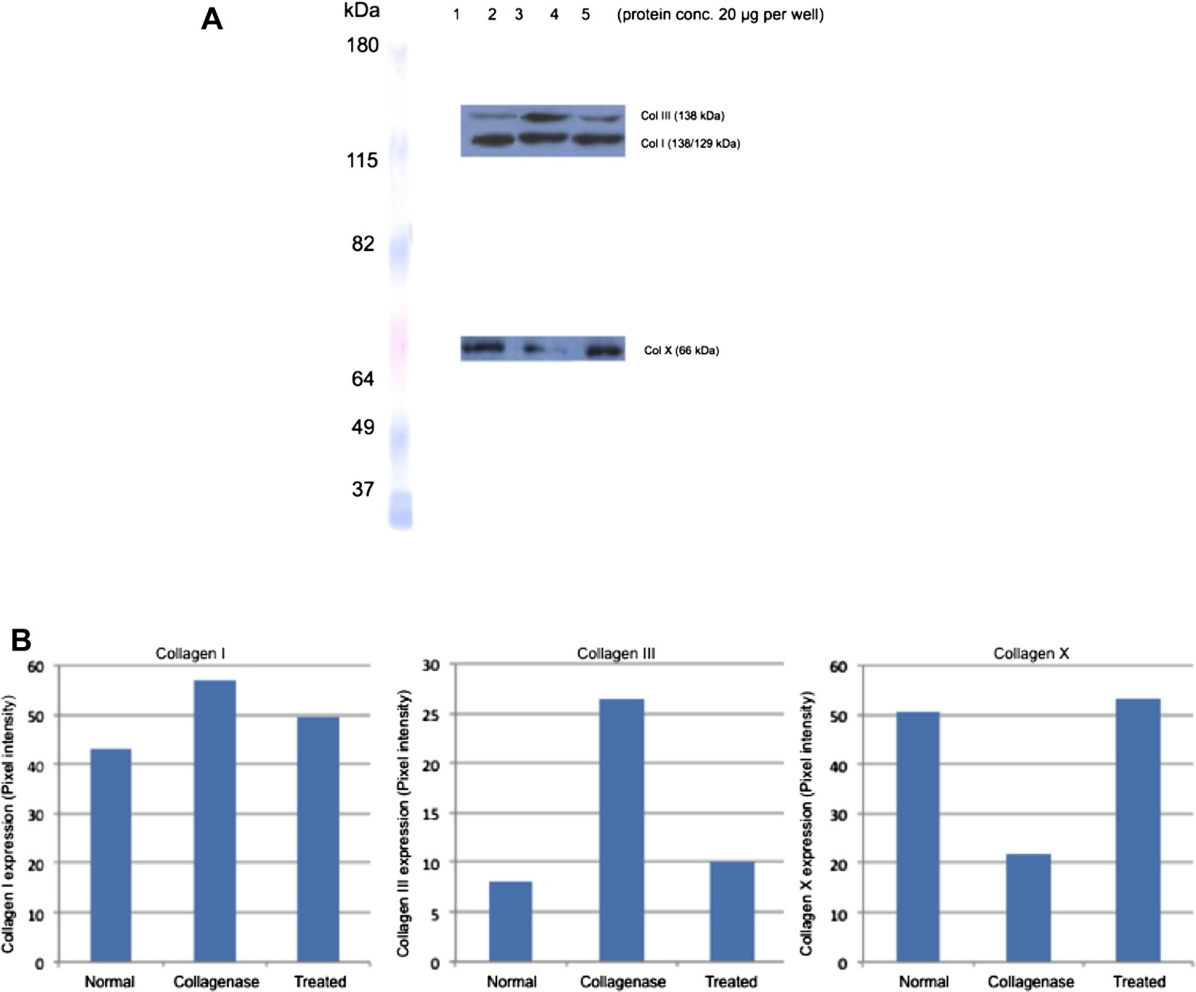
**a** Semi-quantification of collagen subtypes using western blot analysis. **b** The *Y*-axis corresponds to signal intensities: (*A*) collagen I (129 kDa), (*B*) collagen III (138 kDa), and (*C*) collagen X (66 kDa)

As shown in Fig. 4, the collagen content of types I, III, and X varied significantly between the control, tendinopathic (group I), and treated (group II) groups. Although there is a mild elevation in the levels in the treated group (group II) compared to the controls, no statistically significant difference was demonstrable. Collagen I was noted to increase 30 % (±5 %) following collagenase treatment and remained elevated by 16 % following ultrasonic treatment. Collagen III increased 225 ± 30 % following collagenase treatment and remained elevated by 25 ± 5 % following ultrasonic treatment. Collagen X expression dramatically decreased by 58 ± 8 % following collagenase treatment but remained mildly elevated by 4 ± 1 % following ultrasonic treatment. Group 0 was also noted to have changes in collagen expression at 3 weeks following collagenase injection; collagen I increased by 18.4 %, collagen III by 134 %, and collagen X decreased by 10.3 %.

## Discussion

Our results corroborate the use of collagenase to effectively induce a tendinopathic lesion in the Achilles tendon of rabbits, although we wholly understand that there are several other models that produce more physiological tendinopathies. This finding agrees with other studies on tissue repair in the rabbit and with reports on the treatment of tendinopathy [8, 20, 22]. To our knowledge, a study applying ultrasonic therapy to animal model-based tendinopathic tissues has not been reported previously.

The Movin scoring system was used to classify the histopathological findings of the tendinopathy [23]. The assessment system used was semi-quantitative. We are conscious of the limitations of this assessment system, as we categorized in four classes (from 0, normal, to 3, markedly abnormal); a qualitative evaluation of several aspects of the histopathological appearance of the tendon section was examined. It is likely that the fully automated image analysis systems used in other fields of musculoskeletal medicine will be useful in this field in the future and thus allow a more objective quantification of the abnormal appearance of tendinopathic tendons. The Movin score is based on semi-quantitative criteria to assess the changes associated with the process of tendinopathy on a four-point scale ranging from 0 to 3 [23]. This scale was originally developed for the Achilles tendon (Movin) and the patellar tendon, and to assess the degree of tendinopathy in the rotator cuff and the long head of the biceps tendon [23–26]. In this study, we used a modified Movin score, which describes the tendinopathy of the Achilles tendon.

Fibroblasts were well aligned within the tightly packed and longitudinally arranged collagen fibrils in the normal tendon (Fig. 3a). Six weeks after collagenase injection, the tendons showed more immature fibroblast and mononuclear cell infiltration and a significantly disorganized pattern of collagen fibers. The ultrasonic-treated group at 3 weeks demonstrated a greater amount of mature and immature fibroblasts, less mononuclear cell infiltration, and a better-aligned pattern of collagen fibers, indicating that the tissue was regenerating (Fig. 3c).

The tendons themselves are composed of longitudinally arranged bundles of fibers; blood supply to tendons is poor compared to muscles and other tissues [27, 28]. The neovascularization, histologically demonstrated, leads to an improved blood supply and certainly plays a role in the tissue regeneration.

In this study, there were differences seen between the control, injured, and treated groups, in vascularity, collagen density, and collagen fiber organization (Fig. 3), results of which are in agreement with previous published studies [29, 30].

In accordance with other studies, the proportion of type III collagen was increased in specimens of ruptured Achilles tendon [31]. Type III collagen is a major fibril collagen in compliant tissues such as skin and blood vessels and is normally only found in small quantities of normal tendons [32]. Maffulli et al. reported greater amounts of type III collagen in ruptured and tendinopathic Achilles tendons compared to normal human Achilles tendons [33]. The results from this current study are twofold, in that a massive increase in collagen II following collagenase treatment may demonstrate a greater fragment availability for western blot analysis, while a true up-regulation of 25 % was present at 3 weeks after ultrasonic treatment. The latter result corroborates those previous studies, reinforcing the role of collagen III as an important early stabilizer of a repairing/regenerating tendon [25–27].

Recently, some researchers have mentioned the abundance and ratio of type I and type III collagens [34, 35]. The change of the collagens I to III ratio after administration of reagents and after tendon rupture was highlighted. Thomopoulos et al. studied, a canine model, the effects of exogenous basic fibroblast growth factor on intra-synovial flexor tendon healing [35]. Tendons that were treated with basic fibroblast growth factor had a lower ratio of type I collagen to type III collagen from DNA concentration. This indicated increased scar formation due to the growth factor. Otoshi et al. studied the process of tendon regeneration in an Achilles tendon resection rat model as a model for hamstring regeneration after harvesting for anterior cruciate ligament reconstruction [34]. Using immunohistochemistry, the type I–type III collagen ratio in the regenerate tendon was significantly decreased in the early phase but gradually increased with time. The increase in type III collagen expression would have an influence on the inferior mechanical properties of the immature regenerate tendon. In our study, based on the percentage increase from a control starting point, there was a relative up-regulation of collagen III (25 %) compared to collagen I (16 %), further demonstrating an immature collagen ratio for the regenerating tendon.

In this study, it is interesting to note that collagen X is expressed and mildly up-regulated in the 3-week regenerating tendon. There might be morphological changes similar to tendon insertion, spur formation, intra-tendinous calcification, and fibro-cartilaginous zone [36, 37]. We hypothesized that this cartilage-specific collagen would be similar between the groups, but in the collagenase-treated group, the expression of type X collagen was significantly decreased, as compared to the treated groups. This effect may result from the non-specific collagenase destruction of this protein or a secondary loss due to collagen I disintegration.

Several limitations are recognized with this current study. Although validated, the collagenase-induced tendinopathy model may not truly represent an in-vivo chronic tendinopathy, with several other mechanically induced tendinopathy models available, and as mentioned previously. However, we recognize that all such models have limitations. Secondly, the follow-up time frame after treatment was insufficient to demonstrate a full restoration of the tendon, which will be the subject of future, longer term studies, as well as investigating the effect in different models of tendinopathy. Group 0 consisted of two specimens (to confirm the development of a tendinopathic lesion) compared to four specimens in groups I + II, and while this should be noted, future studies will aim to have equal numbers in all groups. Finally, our power analysis indicated that we needed four specimens per experimental group, but since there was a learning curve involved in the use of the ultrasonic probe, we decided to double the number in group II. This can be viewed as a deviation from an experimental protocol, and future experiments should utilize the same specimen numbers in all groups.

## Conclusions

Two major effects of ultrasonic emulsification and evacuation treatment of a tendinopathic lesion have been identified. Firstly, the treatment results in the removal of pathological degenerate tendon material leaving a debris-free space that becomes filled with cells involved in tendon regeneration. Secondly, the qualitative collagen profile of the tendon is returned to a more normal state. Longer term studies are required to better elucidate the potential for complete tendon healing.
